# Supplementary material for: Tectonic and climatic drivers of Asian monsoon evolution
Source: Nat Commun. 2021 Jun 29;12:4022. doi: 10.1038/s41467-021-24244-z (PMC8242090; doi:10.1038/s41467-021-24244-z)
Supplement: Supplementary file 1 — Supplementary Information [file 41467_2021_24244_MOESM1_ESM.pdf]

Supplementary material for

Tectonic and climatic drivers of Asian monsoon evolution, Thomson J.R et al.

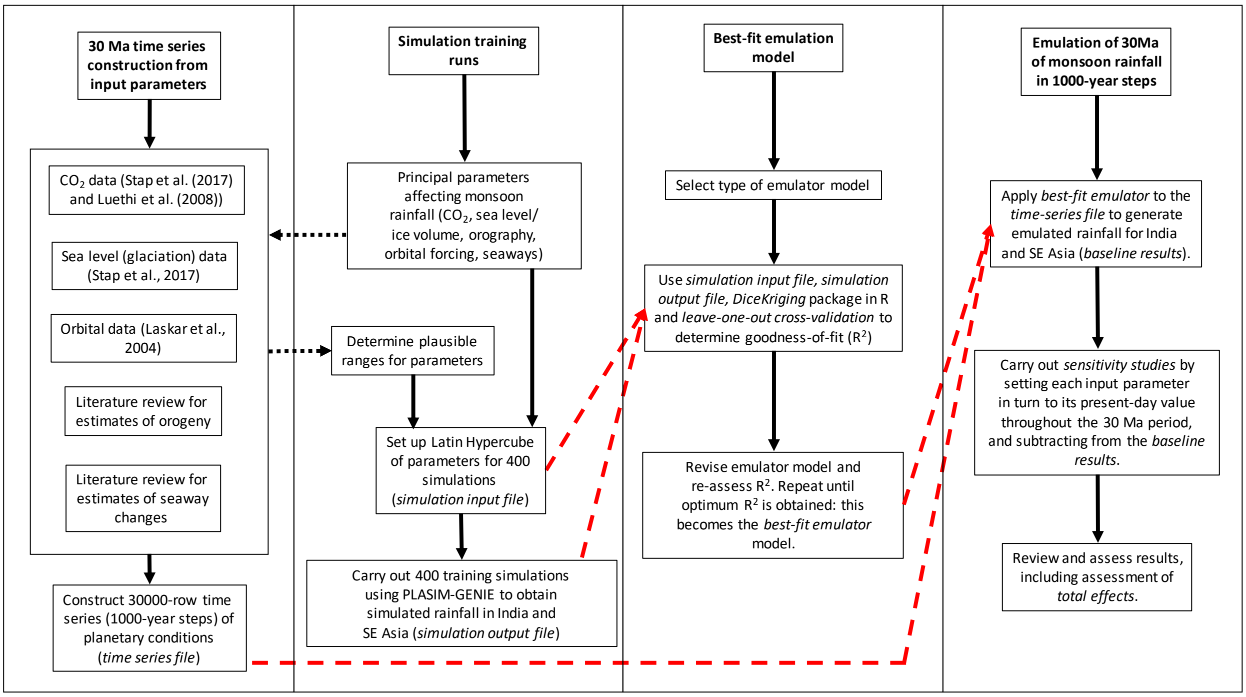

Supplementary Fig.1: Flowchart illustrating methods used.

	30Ma to pre-industrial	Holo-cene	Pleisto-cene	Pliocene	Miocene	Late Miocene	Early Miocene	Late Oligo-cene
		11-0ka	2.58Ma - 12ka	5.333 - 2.58Ma	23.03-5.33Ma	15.97-5.33Ma	23.03-15.97Ma	30.0-23.03Ma
CO_2 SD	143	7	33	42	120	126	76	89
CO_2 mean	482	269	245	308	509	467	571	571
sea level SD	30	12	23	15	21	22	18	19
sea level mean	20	-13	-42	-4	26	21	35	35
precession mvelp ω min	0	0	0	0	0	0	0	0
precession mvelp ω max	360	360	360	360	360	360	360	360
obliquity SD	0.50	0.56	0.56	0.48	0.50	0.50	0.49	0.49
obliquity mean	23.24	23.33	23.32	23.23	23.24	23.25	23.23	23.22
eccentricity (e) SD	0.0131	0.0134	0.0134	0.0127	0.0132	0.0132	0.0131	0.0130
eccentricity (e) mean	0.0275	0.0285	0.0285	0.0268	0.0278	0.2791	0.02752	0.0269
orography (oro) max	1	1	1	1	0.989	0.989	0.667	0.667
orography (oro) min	0.5	1	1	0.989	0.667	0.667	0.667	0.5
world		1	1	1	2	2	3	3

Supplementary Table 1: Ranges of data values, means and standard deviations (SD) used in assessing Total Effects. Holocene eccentricity and obliquity SD and mean are actually Quaternary data, due to paucity of Holocene data.

1) PLASIM-GENIE boundary condition sensitivity simulations

We performed six snapshot simulations with PLASIM-GENIE in order to better understand the underlying model dynamics. These simulations represent the preindustrial state and five sensitivities, having orography reduced by 50%, CO_2 doubled to 560ppm, Last Glacial Maximum (LGM) ice sheets added, Panama gateway opened and precession in opposite phase.

Sensitivities are presented as Jun-July-August averages to highlight the changes in monsoon dynamics. Exceptions are the model sensitivities S16 and S17, which illustrate the change in annual rainfall for direct comparison with the emulated quantities.

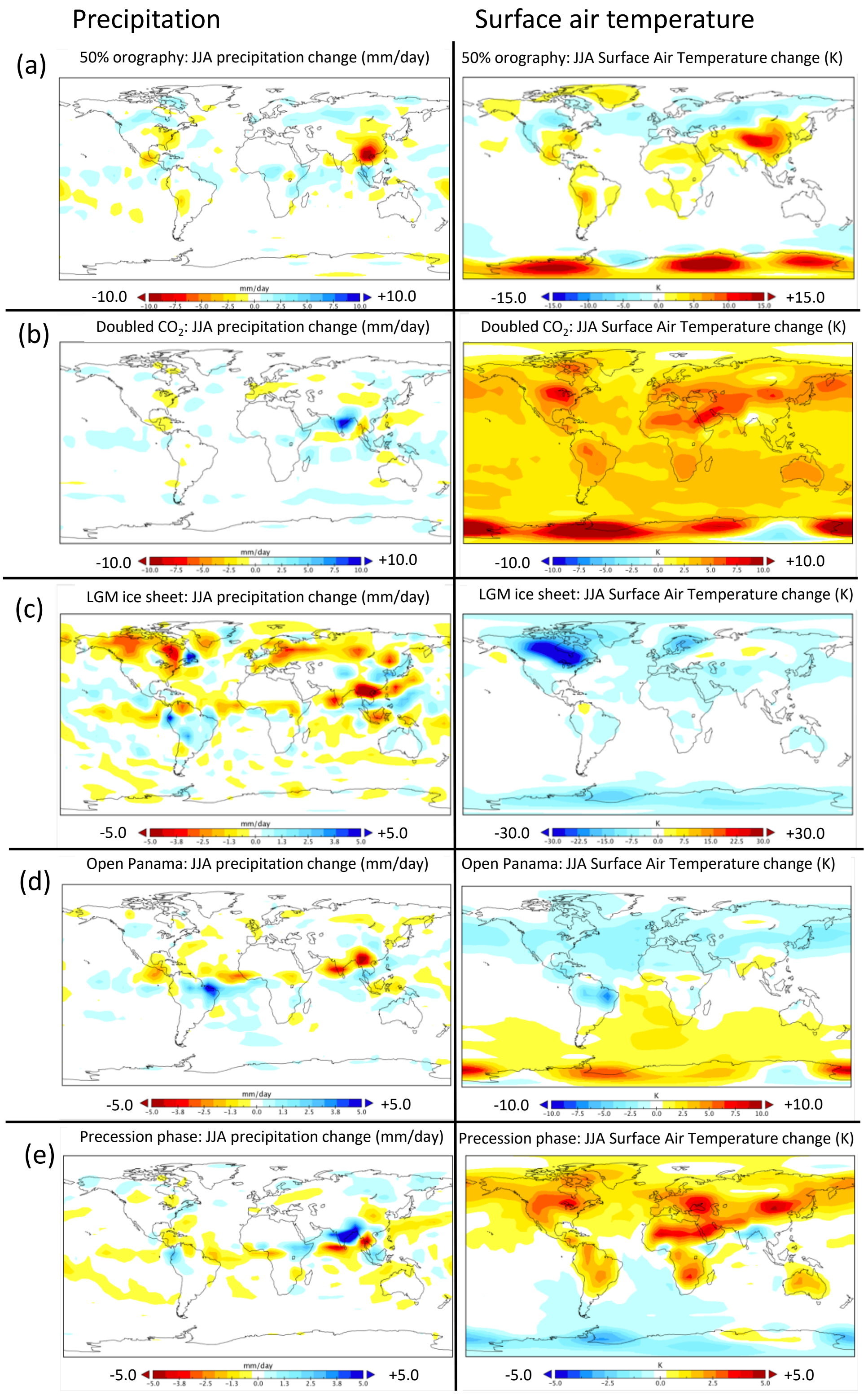

Supplementary Fig.2: PLASIM-GENIE sensitivity simulations for change relative to preindustrial conditions, plotting (left) June July August (JJA) precipitation change and (right) JJA surface air temperature change for (a) 50% orography, (b) doubled CO_2, (c) Last glacial maximum (LGM) ice sheet, (d) open Panama, (e) reversed precession phase. Note the reversed colour scales for precipitation and warming.

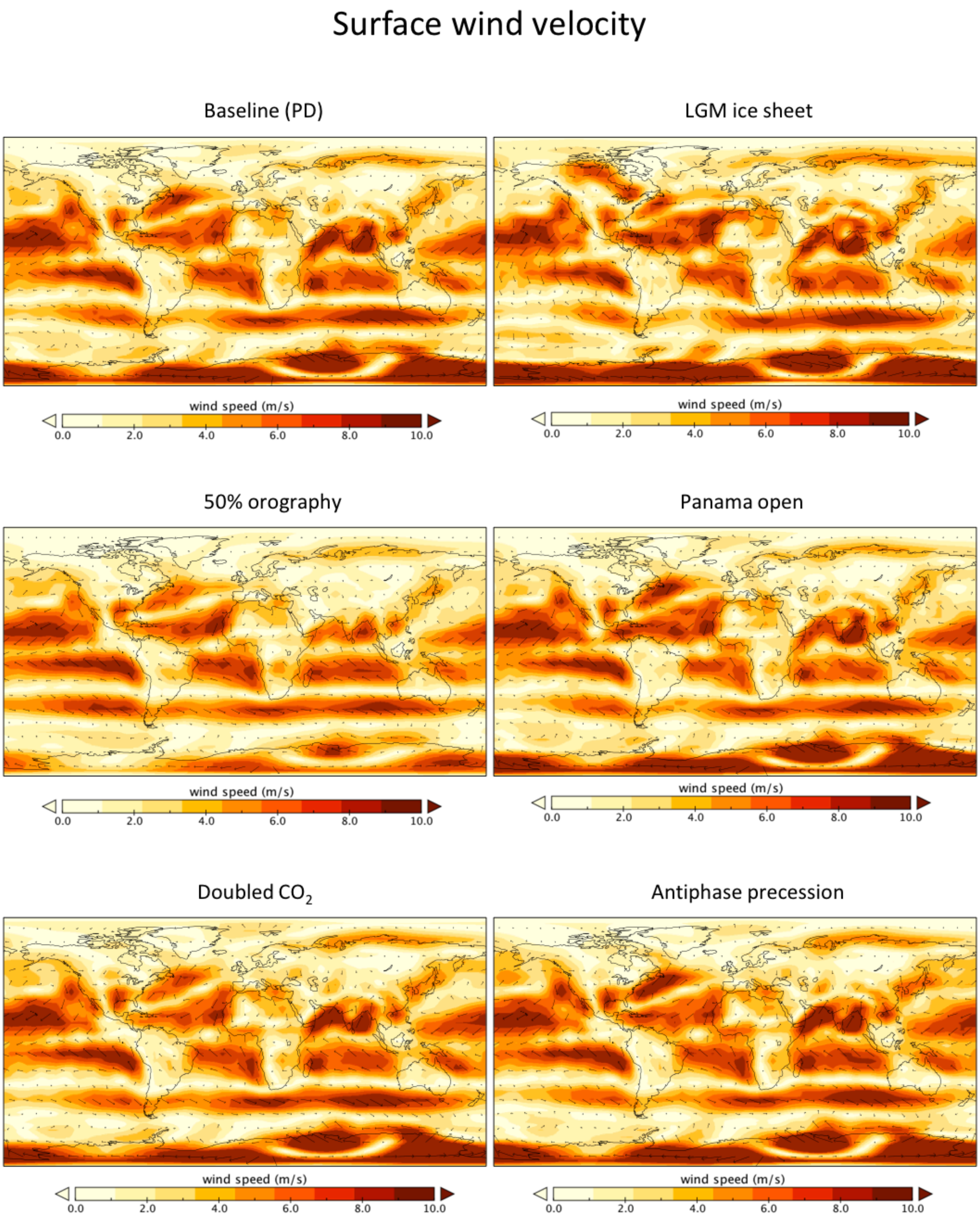

Supplementary Fig. 3: PLASIM-GENIE sensitivity simulations of June-July-August (JJA) surface wind velocity (PD= Preindustrial, LGM=Last glacial maximum)

Hadley circulation

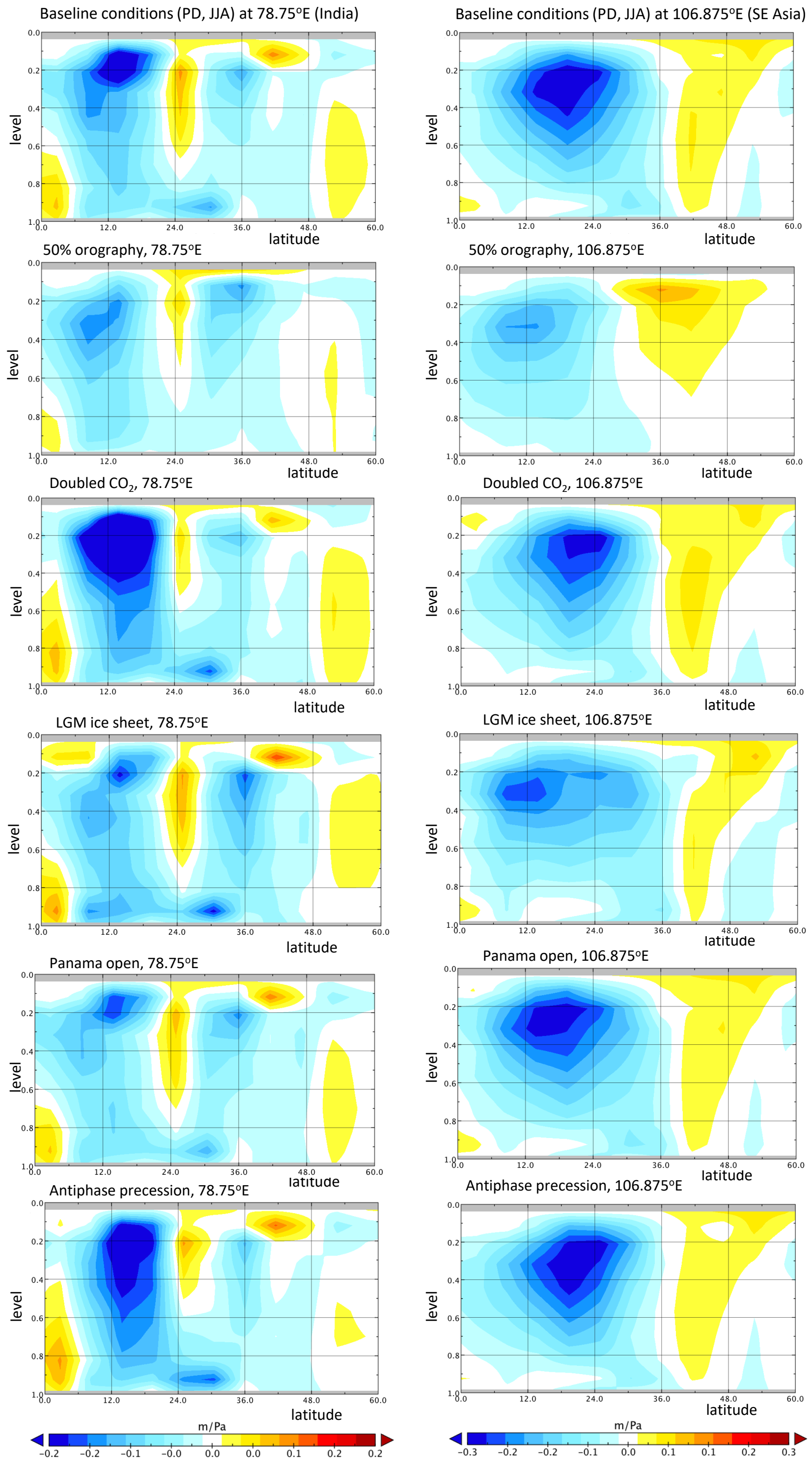

Supplementary Fig. 4: PLASIM-GENIE simulations of June-July-August JJA vertical windspeed to illustrate Hadley circulation. Latitudinal transects from the Equator to 60°N are plotted at longitudes of 78.75°E and 106.875°E in particular illustrating the effect of orography upon SE Asia. (PD=Preindustrial, LGM=Last glacial maximum)

Atlantic meridional overturning circulation

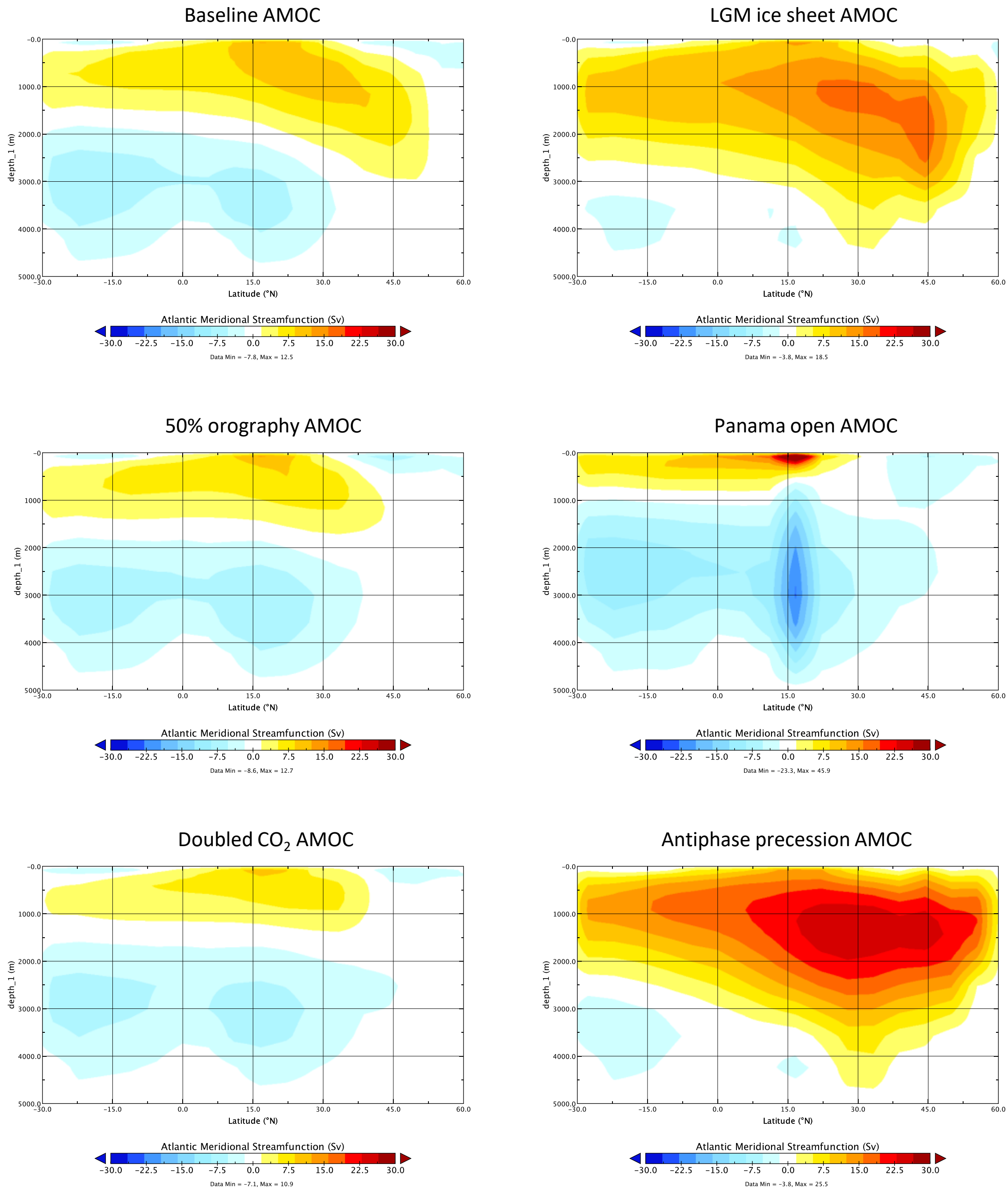

Supplementary Fig. 5: PLASIM-GENIE simulations of Atlantic Meridional Overturning Circulation (AMOC). LGM=Last Glacial Maximum.

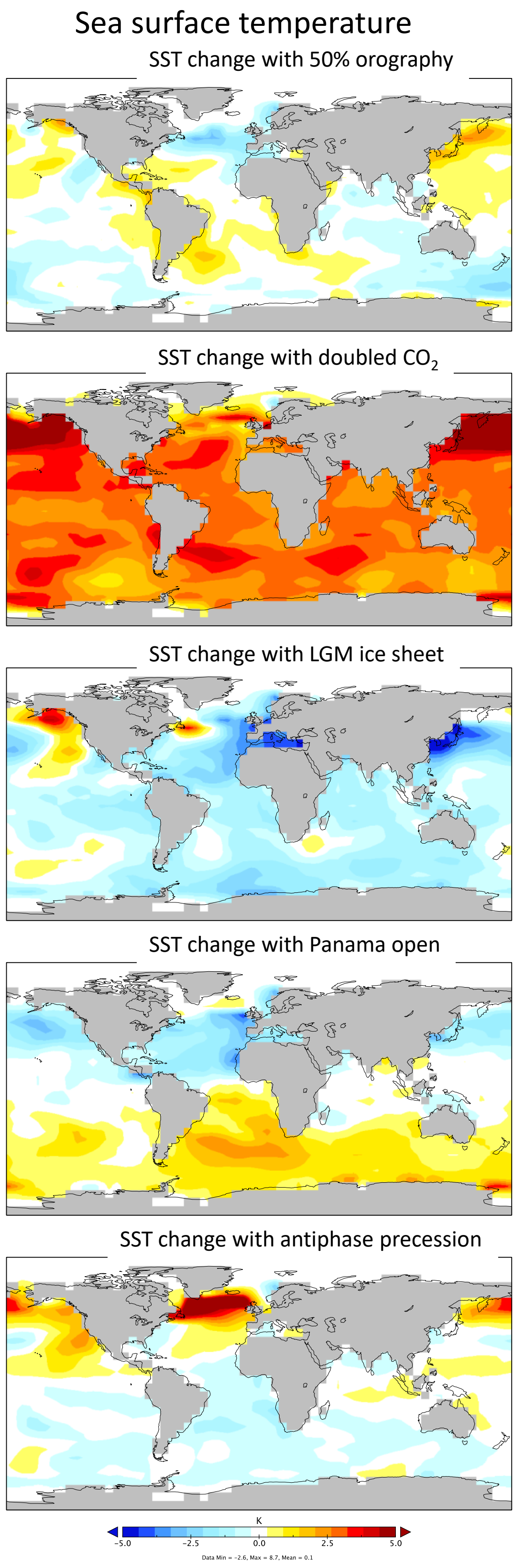

Supplementary Fig. 6: PLASIM-GENIE simulations of sea surface temperature (SST). Changes are relative to preindustrial. LGM=Last glacial maximum.

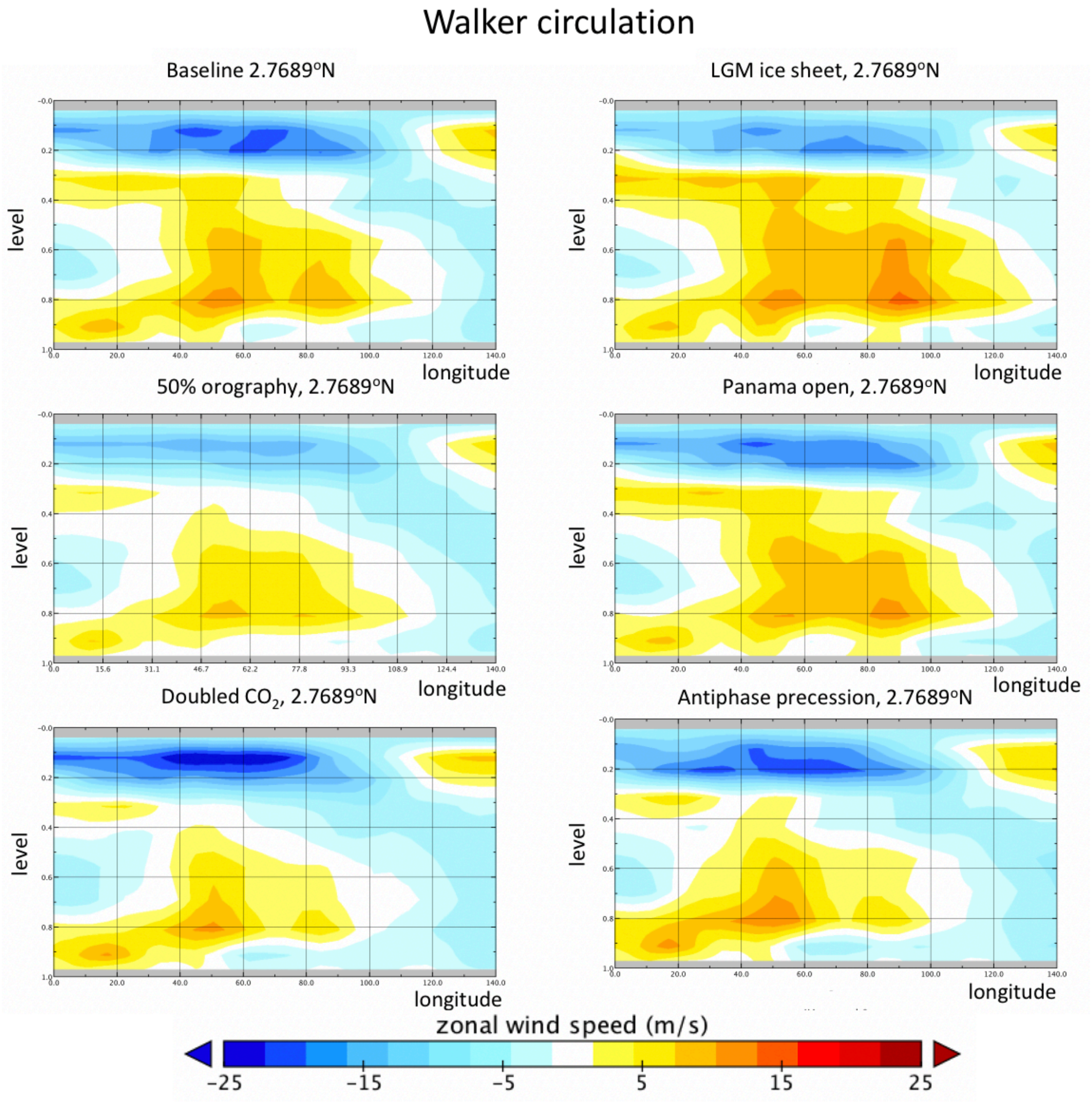

Supplementary Fig. 7: PLASIM-GENIE simulations of June-July-August (JJA) zonal wind speed at 2.7°N to illustrate Walker circulation. Transects run from 0°E to 140°E. LGM=Last glacial maximum.

2) PLASIM-GENIE boundary condition interactions.

We performed eight sensitivity simulations to explore some of the boundary condition interactions. We used the same values of the boundary conditions as the single-parameter sensitivities (Supplementary section 2), but combined them into eight combinations of Tethys open/closed, CO_2 560ppm/280ppm and global orogeny scaling 1.0/0.5.

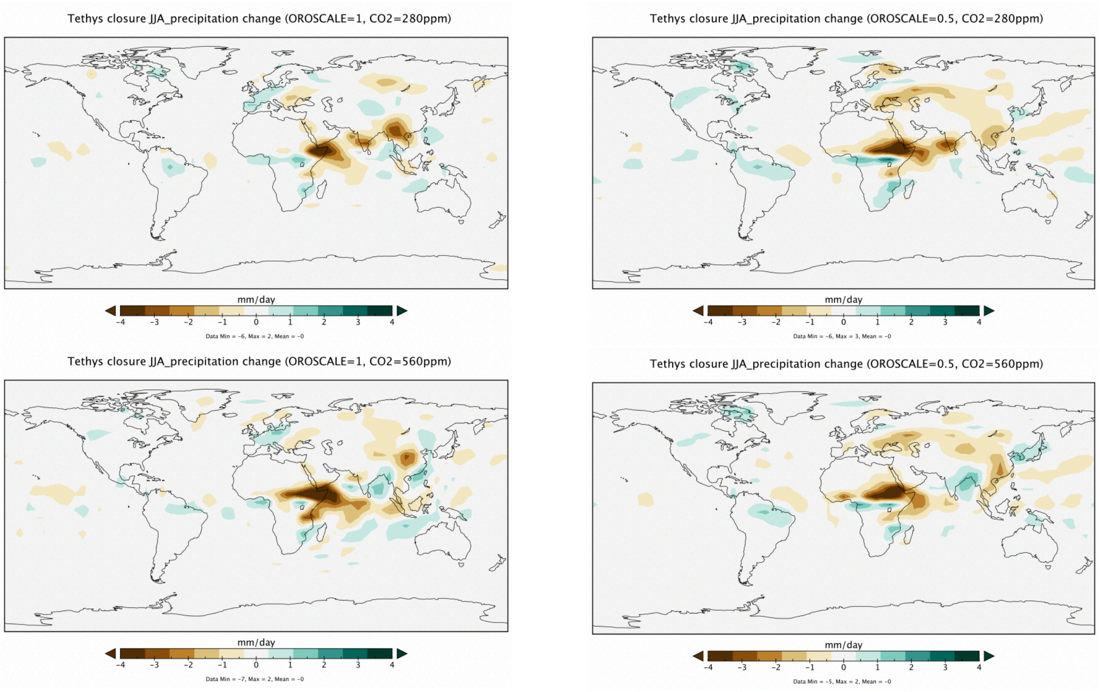

Supplementary Fig. 8: Simulated June July August (JJA) rainfall change on Tethys closure for different combinations of CO_2 and orography forcing. OROSCALE = global scaling of orogeny.

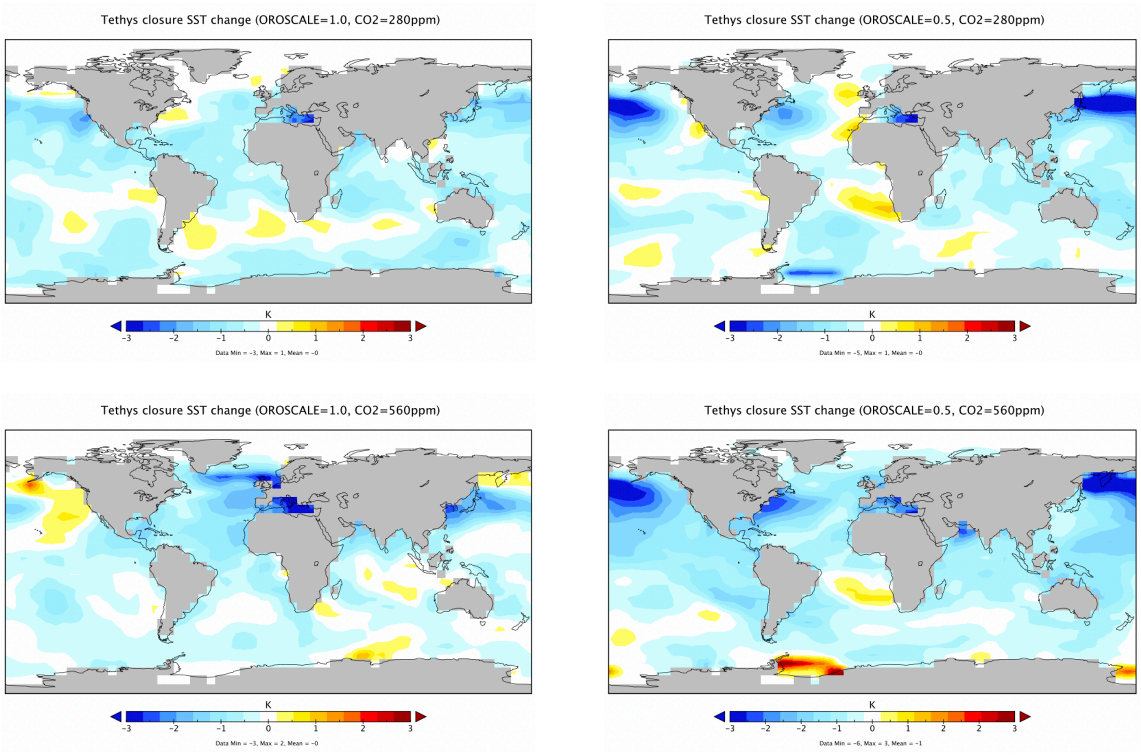

Supplementary Fig. 9: Simulated sea surface temperature (SST) change on Tethys closure for different combinations of CO_2 and orography forcing. OROSCALE=global scaling of orogeny.

3) Late Miocene glaciation synchronized to obliquity cycles

The orbitally-induced rainfall changes during 12-14 Ma shown in Supplementary Fig.10 (a detail from Fig.3d) indicate c. 40 ka-period surges in glaciation. This occurred post-MMCO (Mid-Miocene Climate Optimum), when global cooling was underway, initiated by Tethys closure. The glaciation would have occurred in Greenland[55], with probably also some re-glaciation in West Antarctica. Supplementary Fig.10 indicates that glaciation surges were synchronised with obliquity (with some apparent lagging); this arises because low obliquity means low seasonal contrast and hence cooler high-latitude summers, which reduce the melting of winter snowfall. Also, lesser modulations due to eccentricity cycles are discernible.

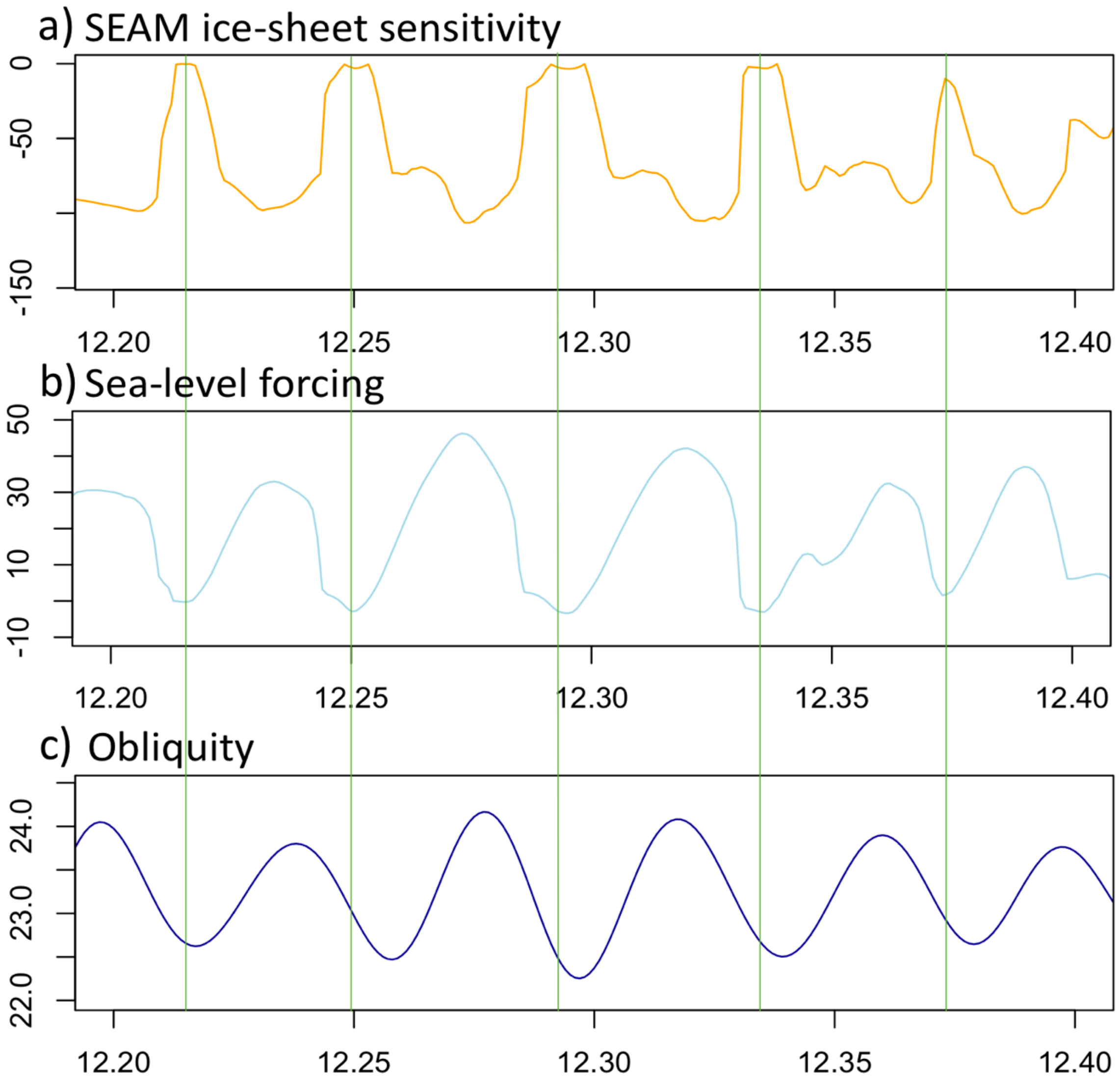

Supplementary Fig.10: (a) Detail from Fig.3d covering 12.2-12.4 Ma, illustrating South East Asian Monsoon (SEAM) rainfall sensitivity to sea level (glaciation) changes. This shows 40 ky-period SE Asia rainfall oscillations (mm/year) during a period of falling pCO_2 and glaciation (from about 15 to 10Ma). (b) Detail from Fig. 2, showing sea-level forcing (m). (c) Detail from Fig. 2 showing obliquity cycles (degrees) synchronised with glaciation cycles. Rainfall peaks (a) coincide with increased glaciation/reduced sea level (b), lagged with respect to obliquity minima (c).

4) Validation of PLASIM-GENIE simulated modern monsoon

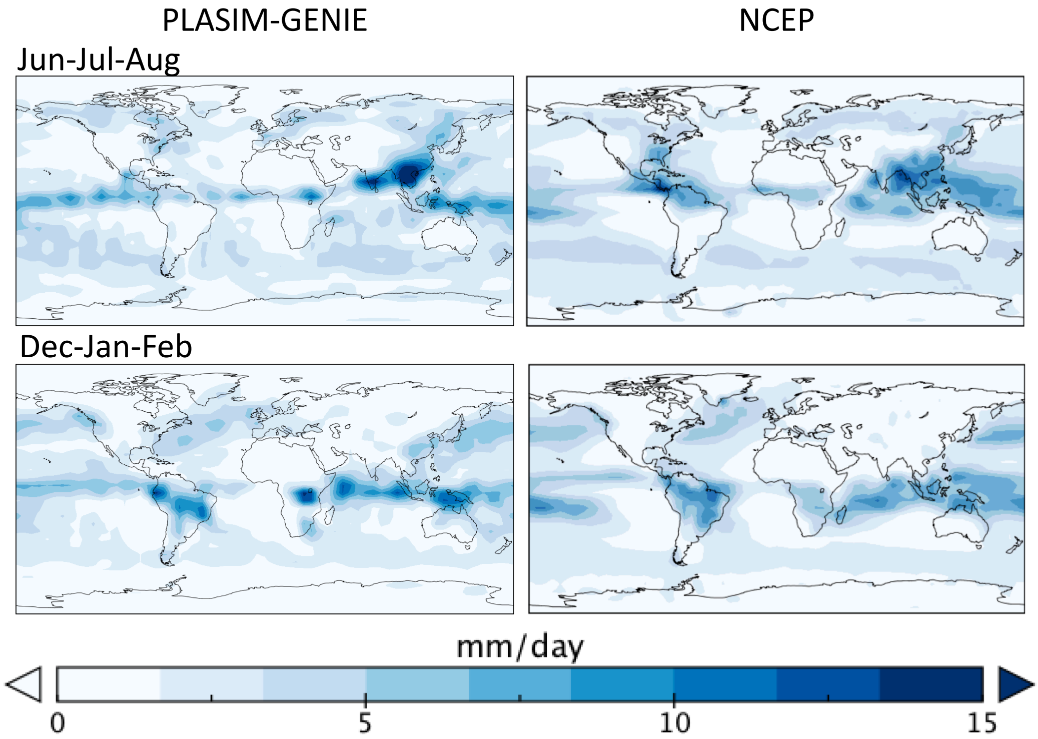

Supplementary Fig.11: Simulated precipitation fields (2005-2015) compared with National Centres for Environmental Prediction (NCEP) reanalysis (Kalnay et al 1996).

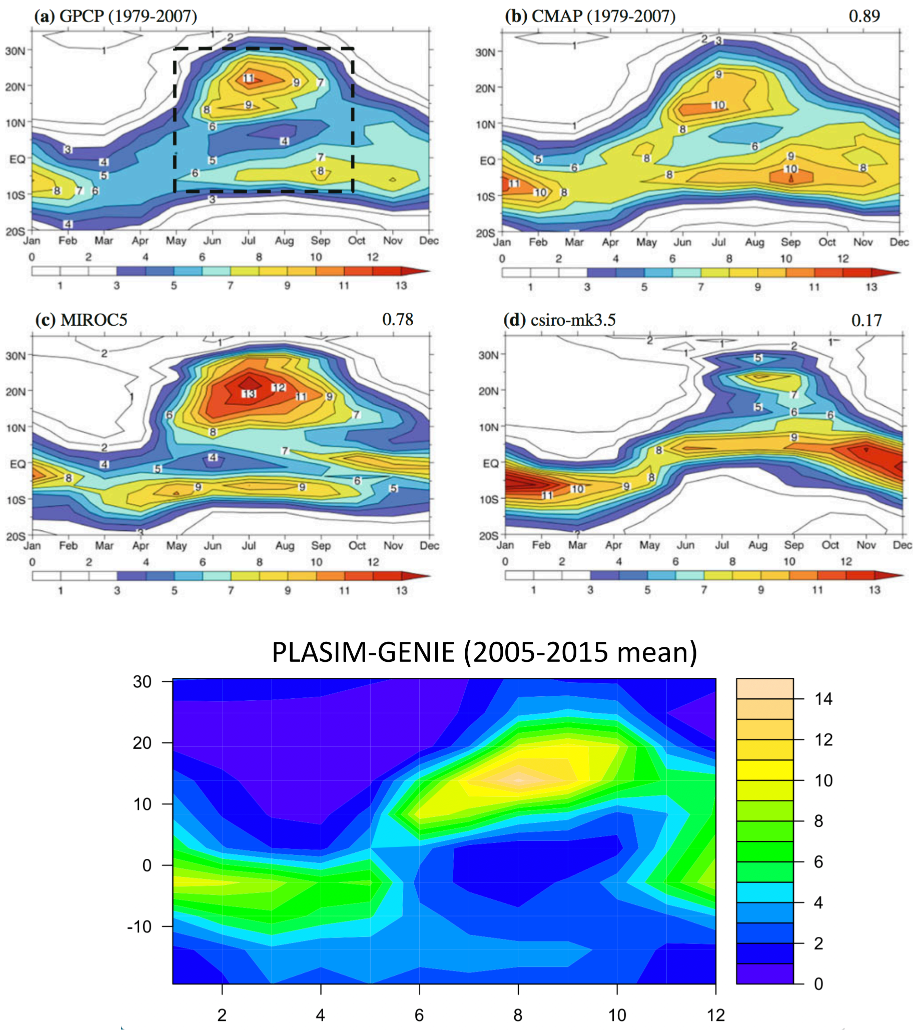

Supplementary Fig.12: The zonally averaged (70°E-90°E) distribution of modern simulated rainfall (2005-2015) through the seasonal cycle (bottom) compared with (top) observations and (middle) high-resolution simulations (Sperber et al 2013). Units are mm/day. GPCP = Global Precipitation Climatology Project. CMAP = Climate Prediction Center Merged Analysis of Precipitation. MIROC5 = Model for Interdisciplinary Research on Climate version 5. csiro-mk3.5 = Commonwealth Scientific and Industrial Research Organisation mark 3.0.

5) PLASIM-GENIE parametric uncertainty

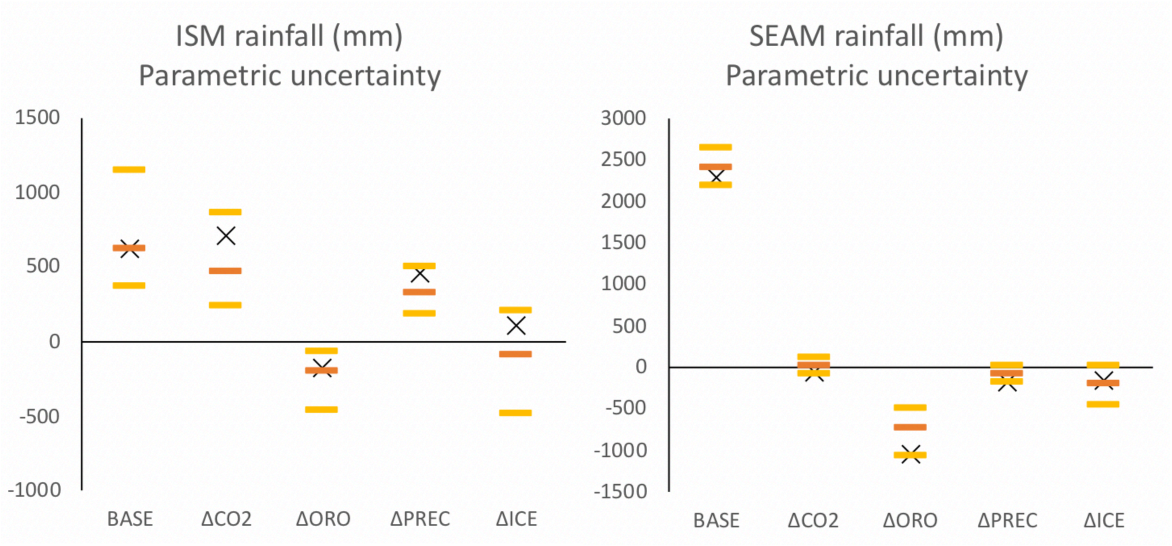

Supplementary Fig.13: Perturbed parameter ensembles using the 69-member pre-calibrated parameter set of Holden et al (2018). These ensembles considered the dominant forcings (Table 1) of orogeny (ORO, global scaling by 50%), CO_2 (doubling to 560ppm), precession (PREC, reversed phase) and last glacial maximum ice sheets (ICE). Orange bars are ensemble medians, yellow bars are the 90% confidence intervals and black crosses are the simulations from the optimised parameter set used to build the emulators. ISM = Indian Summer Monsoon. SEAM = South East Asian Monsoon.

6) PLASIM-GENIE boundary condition uncertainties

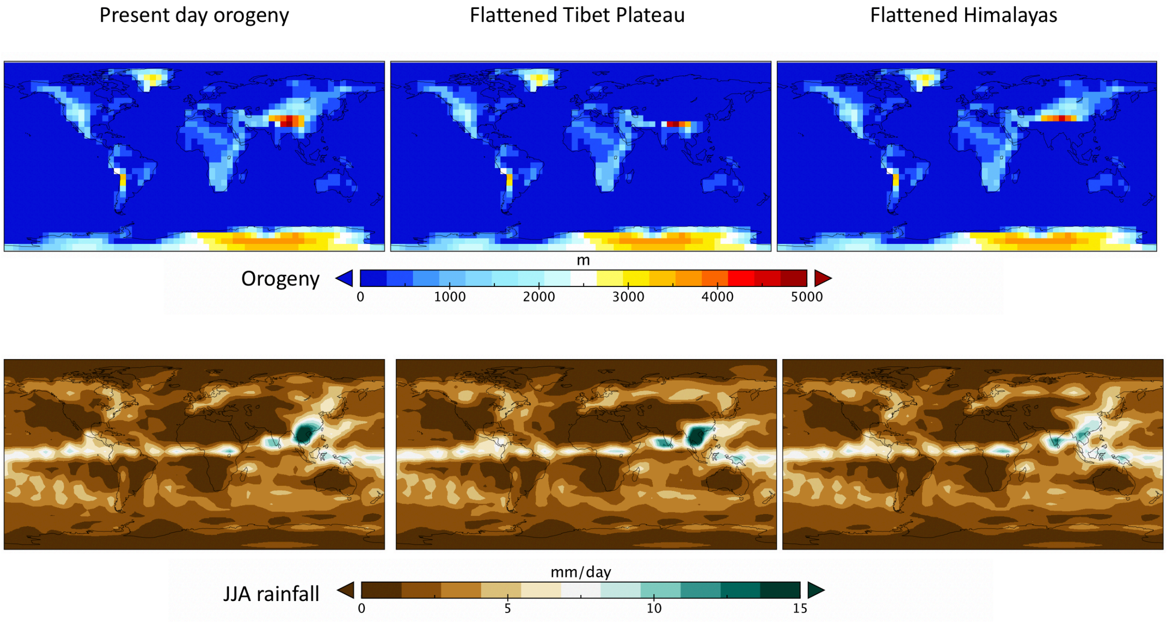

Supplementary Fig.14: The distribution of northern summer rainfall in the baseline preindustrial simulation (left) with simulations that flatten either the Tibet Plateau (centre) or the Himalayas (right). Top row plots the global orogeny assumptions, bottom row plots the Jun-Jul-Aug (JJA) rainfall.

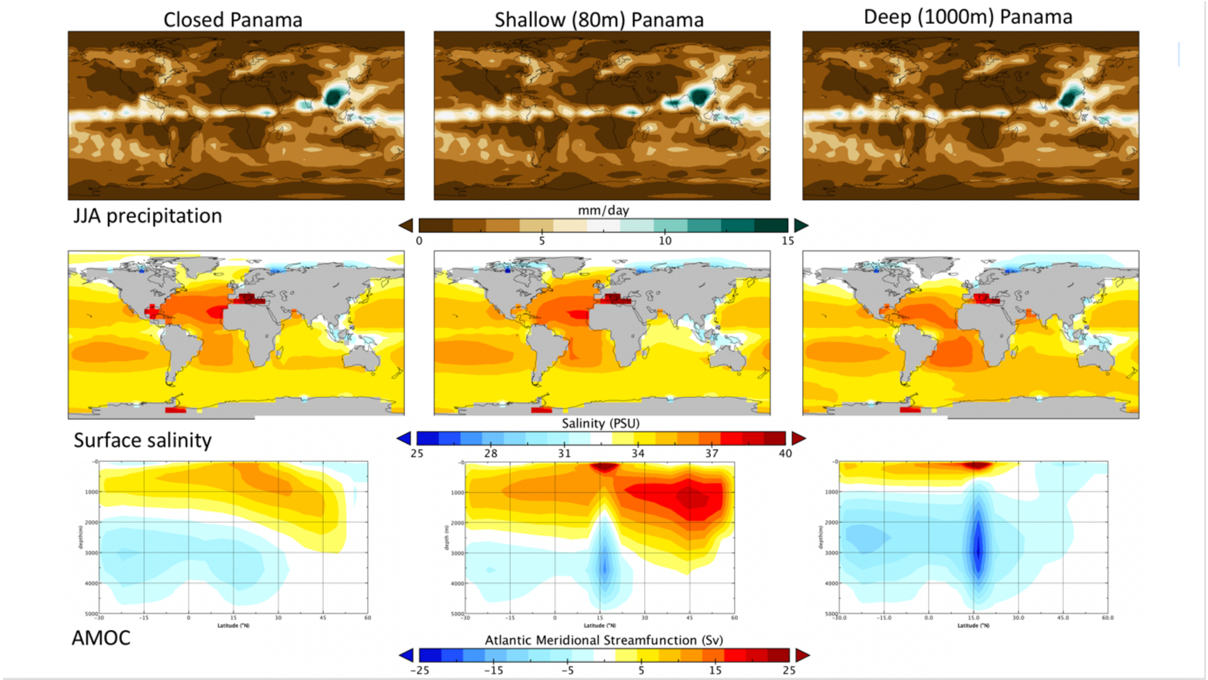

Supplementary Fig.15: Sensitivity to Panama gateway assumption of closed (left), 80m deep gateway (centre) and 1000m deep gateway (right). Fields are Jun-Jul-Aug (JJA) rainfall (top), surface ocean salinity (middle) and Atlantic meridional overturning circulation (AMOC, bottom).

Reduced solar constant (-3.5Wm^{-2}) at 30Ma.

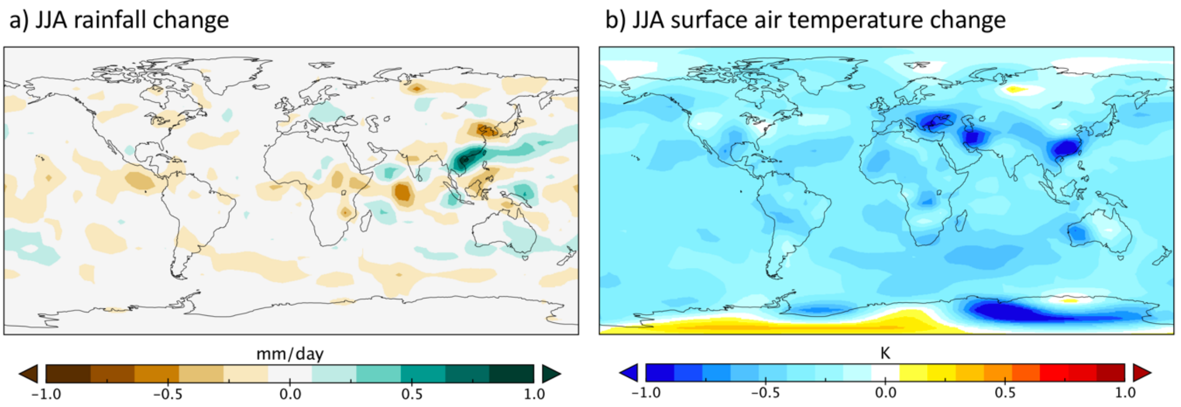

Under a reduced solar constant at 30MA, simulated annual ISM rainfall decreases from 611mm to 583mm. Annual EASM rainfall is unchanged at 1262mm.

Supplementary Fig.16: Changes in a) Jun-Jul-Aug (JJA) rainfall, b) Jun-Jul-Aug surface air temperature and c) annual average rainfall in response to a 3.5Wm^{-2} reduction in the solar constant under 30Ma boundary conditions. Note the legend for annual rainfall (+/- 200 mm), which is an order of magnitude lower than the range used for the dominant drivers (Supplementary Fig. 17). ISM = Indian Summer Monsoon. EASM = East Asian Summer Monsoon.

7) PLASIM-GENIE annual average rainfall sensitivities

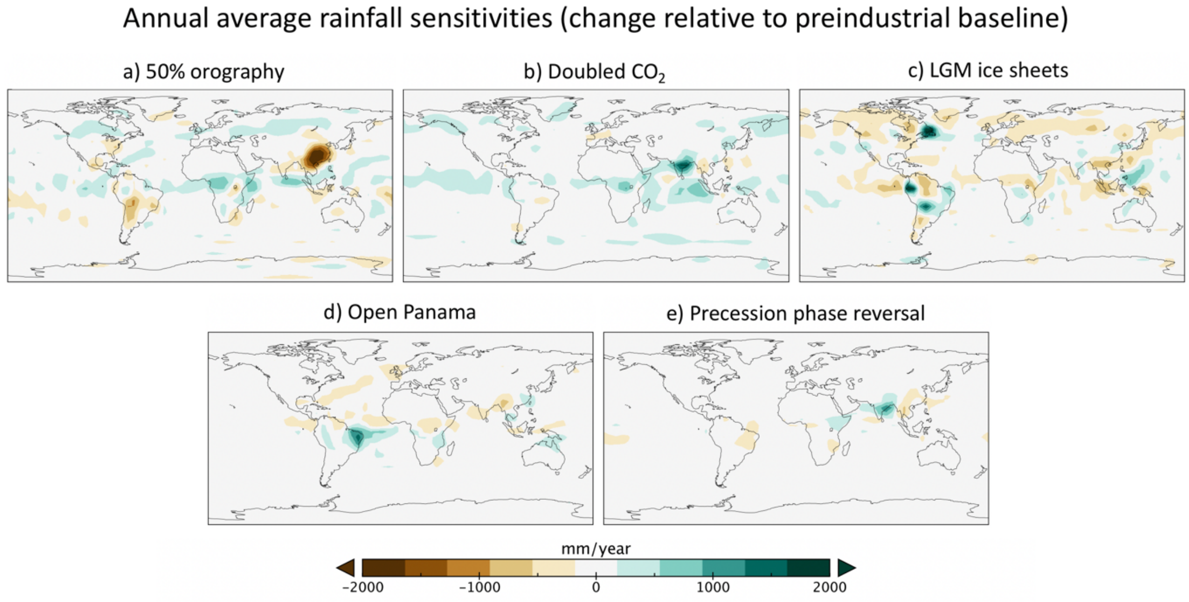

Supplementary Fig.17: PLASIM-GENIE sensitivity simulations for change relative to preindustrial conditions, plotting annual precipitation change for (a) 50% orography, (b) doubled CO_2, (c) LGM (Last Glacial Maximum) ice sheet, (d) open Panama, (e) reversed precession phase.

8) Emulator boundary condition time-series sensitivities

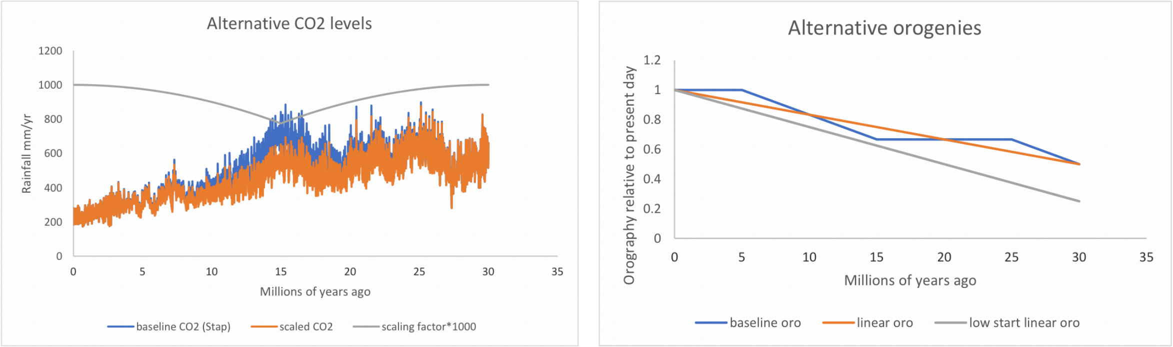

Supplementary Fig.18: Emulator input sensitivities for CO_2 (left) and orogeny scaling (right). The CO_2 scaling is implemented to reduce the Mid Miocene Climate Optimum CO_2 to 560ppm.

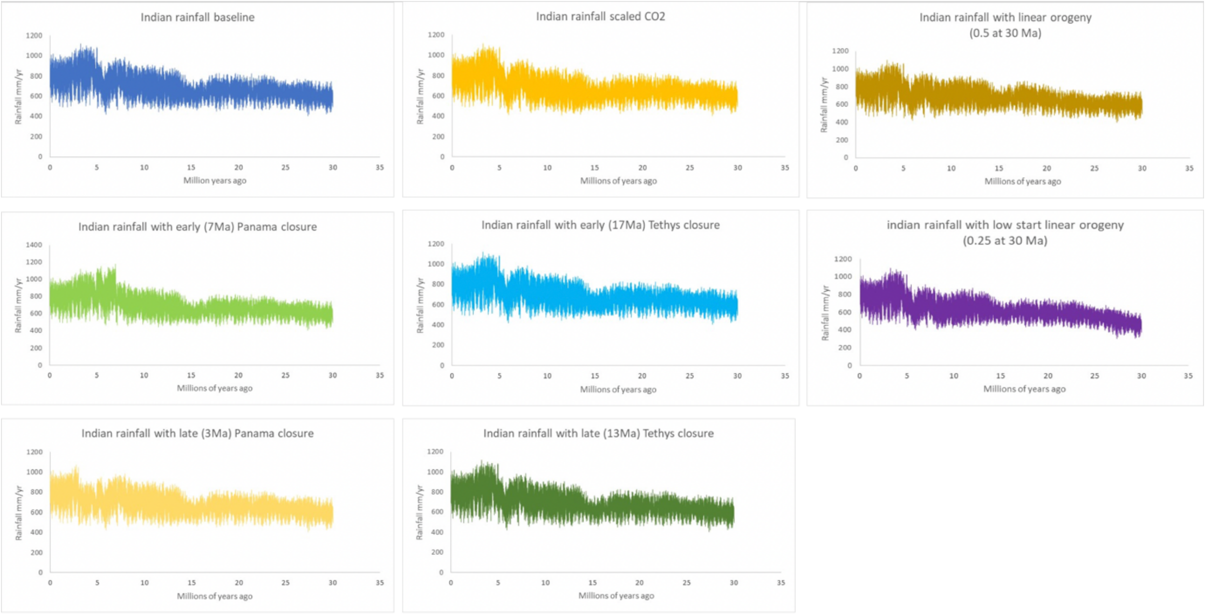

Supplementary Fig.19: Indian summer monsoon emulator sensitivities to alternative boundary condition timing assumptions.

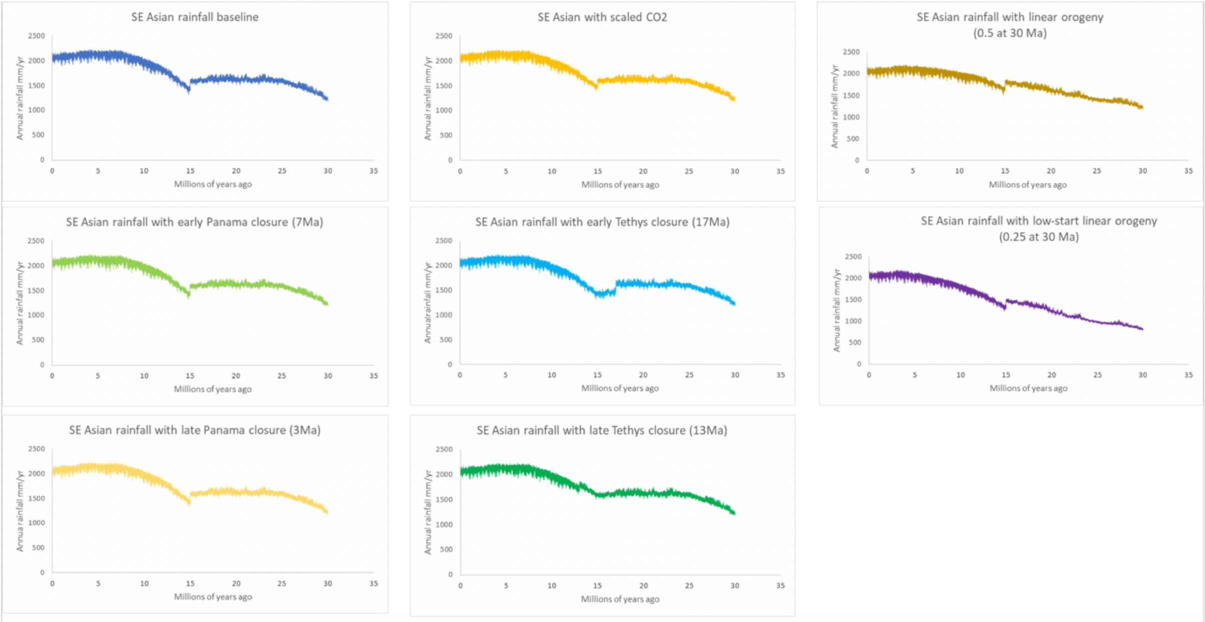

Supplementary Fig.20: South east Asian monsoon emulator sensitivities to alternative boundary condition timing assumptions.
